# The Medical Examination of Employees

**Published:** 1908-06-20

**Authors:** 


### The Hospital

A Journal of

The Medical Sciences and Hospital Administration.

New Series. No. 67, Vol. III. [No. 1139, Vol. XLIV.] SATURDAY, JUNE 20, 1908.

### THE MEDICAL EXAMINATION OF EMPLOYEES.

From time to time within the last two or three years certain suggestions have been made public with regard to the medical examination of various public and private servants with the view to testing their physical fitness for the duties they perform. The last occasion on which such suggestions arose is a sufficiently recent event to be still within everyone's memory, as it was excited by Colonel Yorke's report upon the railway accident near Atherton; but there have been several other similar affairs which have provoked similar comment, notably a recommendation that electric tramcar drivers in the service of the London County Council should periodically be seen by a medical examiner. With the necessity, in the interests of the public or of the employees concerned, of such tests of health we do not propose to concern ourselves: that is a controversial matter on which the medical profession may well be called upon for an expert opinion; but whether such inspection is or is not imperative depends entirely on the merits of the individual occupations and employments, and must be so decided. That which we are considerably more concerned to note is one of the arguments advanced by those who object to all proposals of this kind, and speak with some sort of authority on behalf of those who would be principally affected—that is, by the duly accredited representatives of labour and trades unionism in Parliament and elsewhere. It is said by these objectors that medical examination is degrading, and that to be stripped or partially stripped and examined in the usual way is abhorrent to any decent person, and leads to loss of his self-respect. And this extraordinary proposition has been allowed to pass with little or no comment, though doubtless it did not greatly impress those under whose consideration the suggestions and the objections came. Still the statement was put forward and duly appeared in the press, nor was any reply of suitable vigour reported. It seems to be, therefore, a matter upon which a strong protest is necessary.

There ought not to be, and we do not believe that, in fact, there is anything "degrading" to any sane person in being made the subject of a medical inspection. Every soldier and sailor must submit to it on enlisting in the national forces, whether regular or auxiliary; every civil servant from the highest grade to the lowest undergoes a similar ordeal. Even, in some cases, the very men whose inspection is alleged to be so detrimental have already been medically examined on first taking up their employment. To maintain that the periodical re-investigation of their condition in order to make sure that they are still competent to carry out responsible duties is any more conducive to diminished self-respect than the original test for the same purpose, is so absurd that it only needs to be stated in black and white to be disposed of. If any further evidence is wanted finally to quench the idea that this "degradation" is inseparable from medical examination, it need only be pointed out that almost every man who insures his life is thus degraded, and so are some thousands of private and hospital patients daily throughout the kingdom.

If, then, there need not and ought not to be anything at all objectionable about this very simple and common process, the only other point to consider is whether in actual practice there is or is not anything of the sort—whether, that is, the ordinary medical practitioner or railway medical referee is a person of deliberate vulgarity and inconsiderateness who does his best to convert a medical examination into an inquisition worthy of a mediæval heretic-hunter. To this we can only reply that we do not for a single instant believe that it is usually, or indeed ever, the case; and that if the contention is really implied it is one which ought to be fully and completely substantiated. Until such substantiation of vague allusions and innuendoes is offered we shall firmly decline to believe in the alleged degradation caused by a medical inspection. To put the matter on lower grounds, it is not even as if there was anything to be gained by unnecessary brusqueness or brutality. No business firm, let alone railway companies or public authorities, has any interest in getting rid of valuable, highly skilled, and highly trained servants whose physical condition is fully adequate to their important responsibilities; and therefore no medical man, however unprincipled, can be supposed to have any interest in rejecting the fit or disgusting them into resignation. When, therefore, we find Mr. Hudson asking the President of the Board of Trade in Parliament on May 21 "whether he is aware of the irritating conditions from which the men suffer in connection with medical examination," we are entitled, for the honour of the profession, to demand something more tangible than this rhetorical questioning. For we do not believe that medical examination need carry with it either "irritation" or the "degradation" of which the London County Council were told a few months ago. If it can be proved that it has ever done either, then the fault must be that of some individual medical men, or else that of the bodies by whom they are instructed; and the matter should certainly be cleared up. Unless and until we are offered some satisfactory evidence, beyond mere platform eloquence, we shall continue to disregard the clamour as disingenuous and artificial. Such opposition, based on arguments so unreasonable, merely excites in ordinary minds the suspicion that grave constitutional defects are commoner than may be thought in those to whose vigilance and ability we constantly entrust our safety and our lives, and that the real reason for objecting to such tests of physical and mental competence is a doubt about the result of their application.

